# Notch-1/2 receptors and Jagged-1 ligand, but not HERP-1 transcription factor, are immunohistochemically expressed in the epithelial lining of periapical cysts

**DOI:** 10.4317/jced.61008

**Published:** 2024-01-01

**Authors:** Georgia Nikoloudaki, Eirini-Iouliani Basdeki, Nikolaos P. Kerezoudis, Konstantinos I. Tosios

**Affiliations:** 1 Assistant Professor, Schulich Medicine & Dentistry, Western University, London, Ontario, Canada; 2 DDS, private practice, Athens, Greece; 3 Professor and Chairman, Department of Endodontics, School of Dentistry, National and Kapodistrian University of Athens, Athens, Greece; 4 Associate Professor, Department of Oral Medicine, Pathology & Hospital Dentistry, School of Dentistry, National and Kapodistrian University of Athens, Athens, Greece

## Abstract

**Background:**

To further understand the involvement of Notch pathway signaling in the pathogenesis of periapical cyst the immunohistochemical expression of Notch-1 and Notch-2 receptors, Jagged-1 ligand, and HERP-1 transcription factor in the lining epithelium of periapical cysts was investigated.

**Material and Methods:**

Thirty human periapical cysts were immunohistochemically stained with antibodies against Notch-1, Notch-2, Jagged-1, and HERP-1. Epithelial expression of each antibody was correlated with the presence of inflammation in the connective tissue of the cystic wall.

**Results:**

Notch-1 was identified in the basal and suprabasal epithelial cells of 30/30, Notch-2 in 19/24, and Jagged-1 in 27/30 cysts. HERP-1 was detected in scattered subepithelial inflammatory cells, but not in the lining epithelium of cysts. There was no significant correlation between the immunohistochemical expression of each antibody and the presence of inflammation in the connective tissue of the cystic wall.

**Conclusions:**

This immunohistochemical study showed expression of Notch-1/2 and Jagged-1 in periapical cysts that combined with the expression of HES1/5 found in a previous report, are indicative of the activation of Notch an endocrine-paracrine mechanism. Further research on the activity of Notch and other pathways in periapical cysts may contribute both to elucidate their pathogenesis and select molecular targets for future novel treatments.

** Key words:**Odontogenic cyst, radicular cyst, etiology, epithelial cells, Notch, Jagged, HERP.

## Introduction

The pathogenesis of periapical (radicular) cysts has been associated with the proliferation of the epithelial rests of Malassez (ERM) due to persistence of microbial contamination within the root canal system, through a yet unknown mechanism (1). ERM proliferation may be initiated by pro-inflammatory cytokines, inflammatory moderators, and growth factors secreted by host cells during inflammation of the periapical tissues, as well as by endotoxins and microbial cytokines present in apical periodontitis (2).

Notch signaling pathway is an intercellular signaling cascade that regulates interactions between adjacent cells, permitting them to control each other’s function and fate (3,4). This is accomplished through targeting of specific genes involved in cellular events, such as cell viability, proliferation, differentiation, migration, adhesion, angiogenesis, epithelial-mesenchymal transition, and apoptosis (5). Notch controls morphogenesis, homeostasis, and development during embryonic and adult life, and influences cellular fate (6). It is, also, associated with stem/progenitor cells’ maintenance, differentiation, and proliferation (7) and its disfunction has been correlated with neoplasia and congenital diseases (8).

Notch activation is initiated by four Delta or two Jagged ligands on the extracellular membrane of one cell that interact with Notch receptors of an adjacent cell; this trans-interaction releases the Notch Intracellular Domain (NICD) and leads to transcription of many downstream target genes (7). Downstream targets of Notch are HERP and HES family molecules that are released in various cells during development (9).

Notch may help cells that derive from normal or neoplastic odontogenic epithelium to differentiate and proliferate (10). In odontogenesis, it participates in dental stem cells’ regulation and differentiation, as well as the differentiation of ameloblasts and odontoblasts (11). Notch-2 is expressed in the Hertwig’s epithelial root sheath, whose remnants, ERM, are thought to give rise to the lining epithelium of periapical cysts. Expression of Notch in ERM indicates that this pathway may be, also, involved in the pathogenesis of periapical cysts (12).

Notch receptors have been found in the lining epithelium (3) and inflammatory cells (13) of periapical cysts. Meliou *et al*. (3) suggested that Notch is triggered downstream in the epithelial lining of non-inflamed periapical cysts, as they showed expression of the transcription factors HES1/5 that are the final targets of Notch activation.

To further understand the involvement of Notch signaling pathway in the pathogenesis of periapical cyst, the immunohistochemical expression of Notch-1 and Notch-2 receptors, Jagged-1 ligand, and HERP-1 transcription factor in the lining epithelium of periapical cysts was investigated.

## Material and Methods

**Figure 1:**
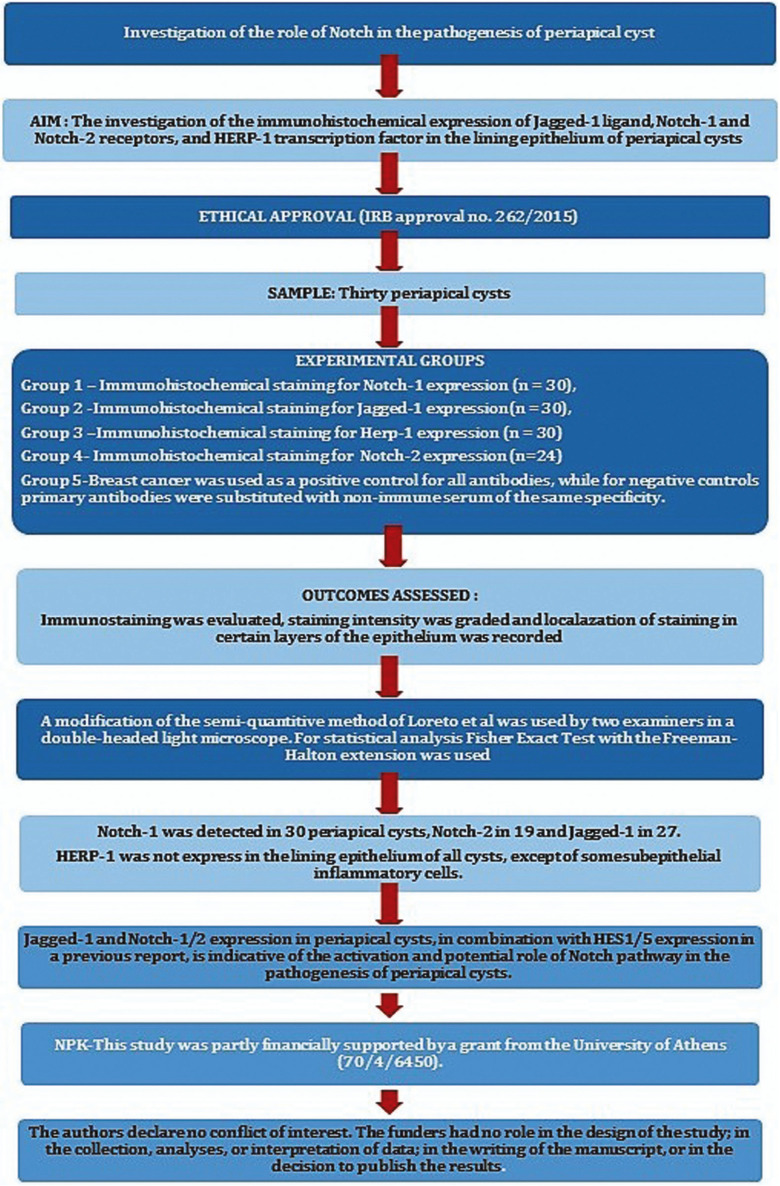
Flow Chart showing the application of Preferred Reporting Items for Laboratory studies in Endodontology (PRILE) 2021 guidelines in the present study.

This study was written in accordance with the Preferred Reporting Items for Laboratory studies in Endodontology (PRILE) 2021 guidelines (Fig. 1). Thirty periapical cysts from the files of the Department of Oral Medicine and Pathology diagnosed between 1990 to 2011, were used in this study (Institutional Review Board-IRB approval no. 262/2015). The tissue specimens were fixed in 10% neutral buffered formalin solution for a minimum of 24 hours and embedded in paraffin. The diagnosis was based on standard histopathological criteria (14). A cyst was classified as non-inflamed or inflamed when inflammatory infiltration occupied ≤20% or >20% of the total space of the connective tissue wall, respectively (15).

Immunohistochemical staining was performed on 5μm thick tissue sections by applying the following primary antibodies: mouse monoclonal Notch-1 (OTI3E12, NBP1-48289, 1:100, Novus Biologicals, Inc., Littleton, USA), rabbit polyclonal Notch-2 (2396-2409- AP07611SU-N, 1:500, Acris Antibodies GmbH, Germany), rabbit polyclonal Jagged-1 (NBP1-90208, 1:100, Novus Biologicals) and rabbit polyclonal HEY1/HERP-1 (ab22614, 1:100, Abcam, Cambridge, UK). For Notch-1 and HEY1/HERP-1 the automated Immunostaining Ventana Benchmark®XT system (Ventana Medical Systems, Inc., USA) was used, and for Notch-2 and Jagged-1 antibodies the Bond Max Automated Immunohistochemistry Vision Biosystem (Leica Microsystems GmbH, Wetzlar, Germany), in accordance with the manufacturer’s protocols. A specimen of breast cancer was used as a positive control for all antibodies, while for negative controls primary antibodies were substituted with non-immune serum of the same specificity.

Jagged-1, Notch-1, and HERP-1 immunohistochemistry was done in all thirty periapical cysts, while for Notch-2 detection twenty-four of the thirty periapical cysts were available.

Immunohistochemical evaluation was conducted on a consensus basis by two examiners, in a double-headed light microscope, by applying a modification of the semi-quantitative method described by Loreto *et al* (16). Staining intensity was graded as 0= faint, 1= moderate, 2= strong, and extent as 0= <5%, 1= 5-50%, 2= >50% of target cells. The final immunohistochemical score was the product of intensity and extent; thus a 4-grade score was utilized, 0=negative staining, 1=mild staining, 2=moderate staining and 4=strong staining. In addition, the localization of staining in certain layers of the epithelium was recorded.

-Statistical analysis

Correlation of immunohistochemical score with inflammation was evaluated using Fisher Exact Test with the Freeman-Halton extension, at *p*<0.05.

## Results

The cysts were excised from 14 female and 16 male patients whose age ranged from 22 to 73 years old (mean age 41 years). Seventeen patients (56.7%) reported no presence of symptoms while thirteen were symptomatic (43.3%). From the total sample, 19 cysts (63.3%) were in the maxilla and 11 (36.6%) in the mandible. The results of the study are summarized in  Table 1.

**Table 1:**
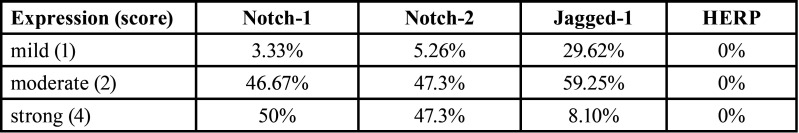
Immunohistochemical score for Notch-1, Notch-2, Jaged-1, and HERP, in periapical cysts.

**Figure 2:**
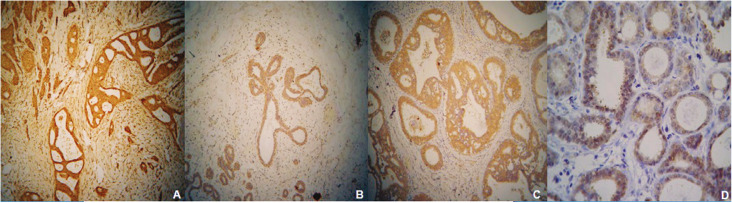
Immunohistochemical expression of Notch-1 (A), Notch-2 (B), Jagged-1(C) and HERP (D) in a mammary carcinoma. No immunoreactivity is present in the stromal cells (immunohistochemical stain with eosin counterstain, original magnifications A-Cx40, Dx200).

Breast cancer cells showed cytoplasmic immunostaining for Jagged-1, Notch-1, Notch-2, and HERP-1 (Fig. 2A-D). There was no immunohistochemical expression in the stromal cells.

**Figure 3:**
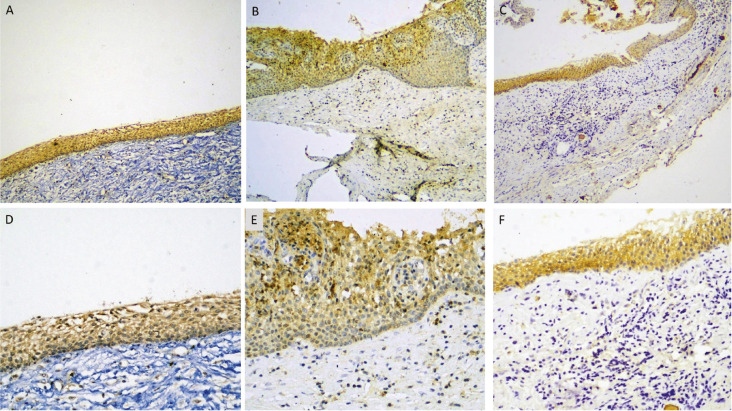
Immunohistochemical expression of Notch-1 (A, D), Notch-2 (B-E), and Jagged (C, F) in the lining epithelium of radicular cysts (immunohistochemical stain with eosin counterstain, original magnifications A-Cx40, D-Fx200).

Notch-1 was detected in the basal and suprabasal epithelial cells of 30/30 periapical cysts (100%), and some subepithelial inflammatory cells, as well as in endothelial cells. One case presented mild staining (3.33%), 14 cases (46.67%) moderate, and fifteen cases (50%) strong staining (Fig. 3 A, D). No significant correlation was found between Notch-1 staining and inflammation (*p*>0.05).

Notch-2 was found in the basal and suprabasal epithelial cells of 19/24 periapical cysts (79.17%), with 5 specimens (20.83%) being negative. There were occasional positive subepithelial inflammatory and endothelial cells. One case showed mild staining (5.26%), 9 cases (47.36%) moderate, and 9 cases (47.36%) strong staining (Fig. 3 B,E). No significant correlation was found between Notch-2 signal and inflammation (*p*>0.05).

Jagged-1 was detected in the basal and suprabasal epithelial cells of 27/30 periapical cysts (90%), with 3 specimens (10%) being negative. Eight cases (29.62%) demonstrated mild, 16 cases (59.25%) moderate and 3 cases (8.10%) strong immunostaining (Fig. 3 C,F). No significant correlation was found between Jagged-1 expression and inflammation (*p*>0.05).

HERP-1 expression was not detected in the lining epithelium of any cyst, while it was seen in some subepithelial inflammatory cells.

Notch-1 and Jagged-1 were co-expressed in 27/30 periapical cysts (90%), while Notch-2 and Jagged-1 were co-expressed in 18/24 periapical cysts (75%) tested for both Notch-2 and Jagged-1. Finally, Notch-1, Notch-2 and Jagged-1 were co-expressed in 18/24 periapical cysts (75%) tested for all three antibodies.

## Discussion

**Figure 4:**
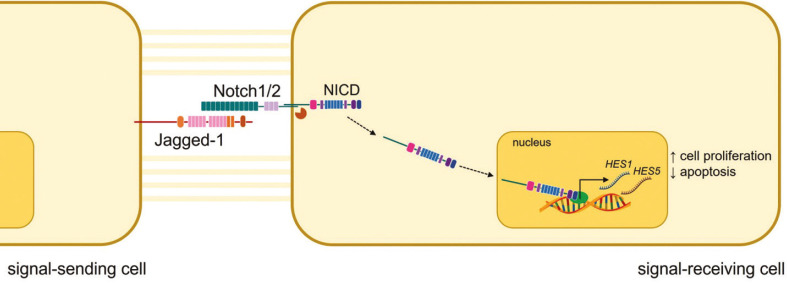
Schematic representation of proposed mechanism of Notch signaling pathway in the epithelial lining of periapical cysts.

The present immunohistochemical study showed that Notch-1/2 receptors and Jagged-1 ligand, but not HERP-1 transcription factor, were expressed in the basal and parabasal layers of the epithelial lining of most periapical cysts. Those findings, combined with the reported expression of HES1 and HES5 transcription factors in periapical cysts (3), indicate that in those lesions Notch signaling pathway is activated (17,18) through the signaling cascade Jagged-1, Notch-1/2, and HES1/5 (Fig. 4). Co-expression of Notch-1/2 and Jagged-1 is indicative of the existence of an endocrine-paracrine mechanism in those cells.

Notch signaling regulates the proliferation of progenitor (7) and mature epithelial cells across a variety of tissues in development, maintenance, and neoplasia (3). Jagged-1 has been correlated with oral carcinogenesis as its levels are progressively increased from oral epithelial dysplasia to infiltrative squamous cell carcinoma (19), while in head and neck carcinomas its overexpression is thought to promote crosstalk between endothelial and tumor cells (19), triggering its activation in endothelial cells and facilitating tumoral angiogenesis and growth (17). High levels of Notch-1/2/3/4 have been identified in breast cancer, where Notch-2 is considered as a tumor suppressor (20), and in cervical cancer in correlation with a poor overall survival (21). Simultaneous expression of Notch-1 and Jagged-1 in breast cancer is thought to stimulate self-renewal of the tumor-initiating population (18), and in head and neck carcinomas to facilitate tumor progression through cell proliferation (17). Notch-1, Notch-2, and Jagged-1 were expressed in basal and parabasal layers in most of the periapical cysts examined in the present study. As those cells behave as unipotent stem cells having the ability of cell division on inflammatory stimulation (12), it is suggested that Notch activation may be involved in cell proliferation.

On the contrary, in normal epidermis Notch participates in differentiation, i.e., overexpression of Notch-1 in the basal cells inhibits proliferation and promotes premature differentiation; however, overexpression in squamous cells induces hyperproliferation of basal cells and epithelial hyperplasia (22). In addition, Notch-1 inhibits apoptosis in T-cells and it influences cell cycle kinetics. *In vivo*, hyperactivity of Notch pathway in mouse breast epithelial cells inhibits cell proliferation and clonal outgrowth, induces to mouse mammary stem cells binding along luminal lineage and to bipotent progenitor cells binding to luminal progenitors, and contributes to the differentiation process (23). In ameloblastoma, downregulation of Notchis associated with squamous and granular differentiation of tumor cells that express Jagged-1 and Notch-1/2/3 (24). Differentiation is caused possibly through interaction with the cyclin-dependent kinase inhibitor p21WAF1⁄Cip1, the Sonic Hedgehog (SHH) signaling molecules, and the Wnt signaling pathway (24).

 Therefore, the presence of Notch-1/2 and Jagged-1 in the basal cells, but not squamous cells, of most radicular cysts in the present study may indicate synchronous proliferation and differentiation, and mark “active” lesions where a balance between those cellular function defines the thickness of the epithelial lining and probably their expansile behavior. Those findings, combined with the hypothesis that Notch can inhibit apoptosis in epithelial cells of periapical cysts (3) can establish an association between Notch and maintenance of the cystic lining epithelium. However, lack of expression of Notch-1/2 and Jagged-1 seen in some periapical cysts may suggest a reduction in their proliferative capacity and mark more “quiescent” lesions.

Notch plays an important role in promoting epithelial-mesenchymal transition (EMT) in cardiac development and oncogenic transformation (25). EMT has been shown in odontogenic keratocysts (26,27), and Notch activation in periapical cysts, as indicated by the findings of the present study, could form a basis for investigating its presence in those lesions.

Notch contributes to the emergence of inflammation, as it regulates the differentiation and activation of cells of the immune system, mostly macrophages, dendritic cells (DCs) and lymphocytes (28). It controls the homeostasis of CD4+/CD8+ lymphocytes, regulatory T-cells, B-cells, natural killer (NK), and dendritic cells (29). It is, also, crucial for the commitment of T-cell and B-cell lineages and primary stages of thymocyte, along with the development of Marginal Zone B-cell (MZB) (30). In synovial fibroblasts of patients with rheumatoid arthritis Tumor Necrosis Factor (TNF) drives the expression of Jagged-2, Notch-1, and Notch-4, along with a hallmark of Notch activation, the Notch intracellular domain (NICD) nuclear translocation (8). In some epithelial tissues, i.e., skin, Notch is a negative regulator of inflammation (31). As no statistically significant association was found between epithelial Notch-1/Notch-2/Jagged-1 expression and the presence of inflammation in our material, it is suggested that inflammation does not exert an effect in the epithelial cells through Notch signaling.

The main limitation of this study is the small number of periapical cysts examined, but the use of immunohistochemistry for the detection of Notch pathway components provides information on the exact cellular and tissue localization of the proteins under investigation. Our findings should be considered preliminary.

In conclusion, this immunohistochemical study showed expression of Notch-1/2 and Jagged-1 in periapical cysts that combined with the expression of HES1/5 found in a previous report, are indicative of the activation of Notch an endocrine-paracrine mechanism. Further research on the activity of Notch and other pathways in periapical cysts may contribute both to elucidate their pathogenesis and select molecular targets for future novel treatments.
